# Introduction and Administration of the Clinical Skill Test of the Medical Licensing Examination, Republic of Korea (2009)

**DOI:** 10.3352/jeehp.2010.7.4

**Published:** 2010-12-03

**Authors:** Kun Sang Kim

**Affiliations:** National Health Personnel Licensing Examination Board, Seoul, Korea.

**Keywords:** Medical Licensing Examination, Clinical Performance Examination, Standardized Patient, Objective Structured Clinical Examination

## Abstract

The first trial of the clinical skill test as part of the Korean Medical Licensing Examination was done from September 23 to December 1, 2009, in the clinical skill test center located in the National Health Personnel Licensing Examination Board (NHPLEB) building, Seoul. Korea is the first country to introduce the clinical skill test as part of the medical licensing examination in Asia. It is a report on the introduction and administration of the test. The NHPLEB launched researches on the validity of introducing the clinical skill test and on the best implementation methods in 2000. Since 2006, lists of subjects of test items for the clinical skill test has been developed. The test consisted of two types of evaluation, i.e., a clinical performance examination (CPX) with a standardized patient (SP) and objective structured clinical examination (OSCE). The proctor (medical faculty member) and SP rate the examinees' proficiency for the OSCE and CPX respectively. Out of 3,456 applicants, 3,289 examinees (95.2%) passed the test. Out of 167 examinees who failed the clinical skill test, 142 passed the written test. This means that the clinical skill test showed characteristics independent from the written test. This successful implementation of the clinical skill test is going to improve the medical graduates' performance of clinical skills.

## INTRODUCTION

The National Health Personnel Licensing Examination Board (NHPLEB) is an institute that presides over 21 medical health personnel licensing examinations including the Medical Licensing Examination authorized by the Ministry of Health and Welfare, Republic of Korea. Beginning in 2009, to become a physician in Korea, candidates should meet two basic requirements and should pass the Medical Licensing Examination. Not only anyone who has graduated from medical schools in Korea and has earned a bachelor's degree in medicine but also anyone who has graduated from any international medical school accredited by the Minister for Health and Welfare, obtained a medical license in that country, and passed a preliminary test before the Medical Licensing Examination is eligible to take the Medical Licensing Examination in Korea. The Medical Licensing Examination consists of a written test and a clinical skill test. To obtain a medical license, an applicant must pass both the written test and the clinical skill test. The written test has been executed in early January every year. Meanwhile, the first trial of the clinical skill test was performed for 3 months from September to early December, 2009, in the clinical skill test center located in the NHPLEB building, Seoul. This report reports on the introduction and administration of the clinical skill test as part of the Korean Medical Licensing Examination in order to share the experience with other countries' examination boards that may wish to adopt a clinical skill test in their health personnel licensing examinations.

## PREPARATION OF THE CLINICAL SKILL TEST

In 2006 it was announced that the integration of the clinical skill test into the Medical Licensing Examination in Korea would begin in 2009. The clinical skill test measures proficiency in clinical skills using a mannequin, models, or various equipment as well as attitude and knowledge with the help of systematically trained standardized patients [1]. The move to change the curriculum of medical schools to enforce bedside teaching and the communication with patients rather than lecture-oriented or recall type education has been gaining momentum. Every medical school in Korea has begun to provide a clinical skill center before the first introduction of the clinical skill test. Furthermore, consortia for training for the clinical skill test were organized in several regions so that active research on clinical skill testing has been possible [2]. The NHPLEB has initiated and supported a research project to test the validity of introducing the clinical skill test and to find the best implementation methods since the year 2000. In March 2007, NHPLEB organized the Committee on Promotion of the Clinical Skill Test, the Committee on Execution of the Clinical Skill Test, and the Committee on the Review of Test Items for the Clinical Skill Test to prepare the test. Since 2006, lists of subjects of test items for the clinical skill test were developed and the precise description of test items was established for the test. Items developed were reviewed again to improve their validity. Also, a simulation test was administered annually since 2006 so that the entire system for the clinical skill test could be evaluated completely. In October, 2008, the clinical skill test center was constructed on two floors of the NHPLEB building.

## ADMINISTRATION OF THE CLINICAL SKILL TEST

### Time table of the clinical skill test

The clinical skill test was announced in July 7, 2009 via the homepage of the NHPLEB (http://www.kuksiwon.or.kr). The duration of receipt of the application was from August 4 to August 8, 2009. The duration of the test was from September 23 to December 1, 2009 (51 days). The release of the list of passing applicants was announced on January 19, 2010 at the same time as the release of the list of applicants passing the written test. The location of the test was the clinical skill test center at the NHPLEB (two clones). The number of applicants was 3,456. The application fee was 510,000 Korean won (500 US dollars).

### Distribution of the date of test and subjects of the test

**Fig. 1:**
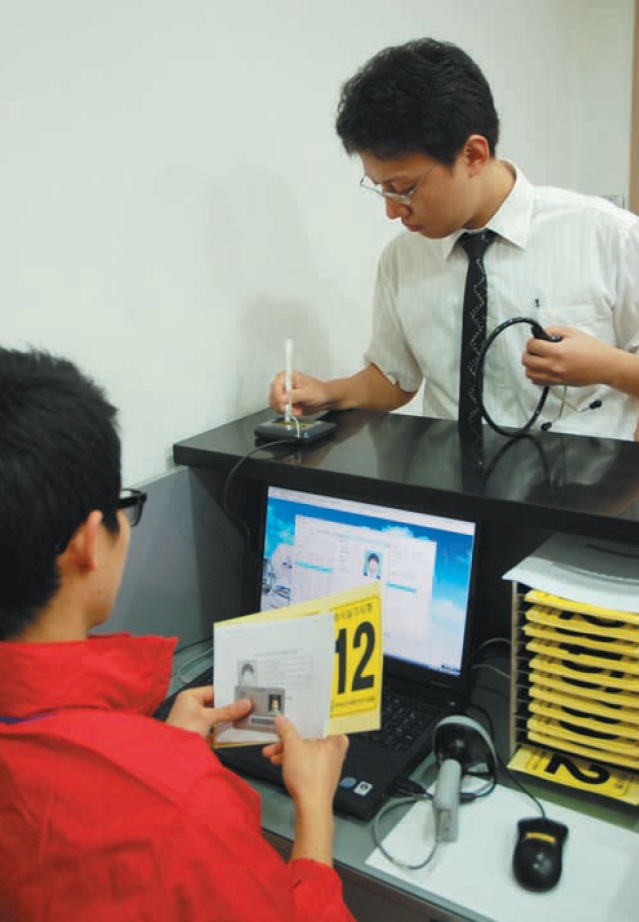
Examinee receives number card to be attached to lab coat after signing. Staff member confirms if the examinee's personal identification is correct before start of clinical skill test.

**Fig. 2:**
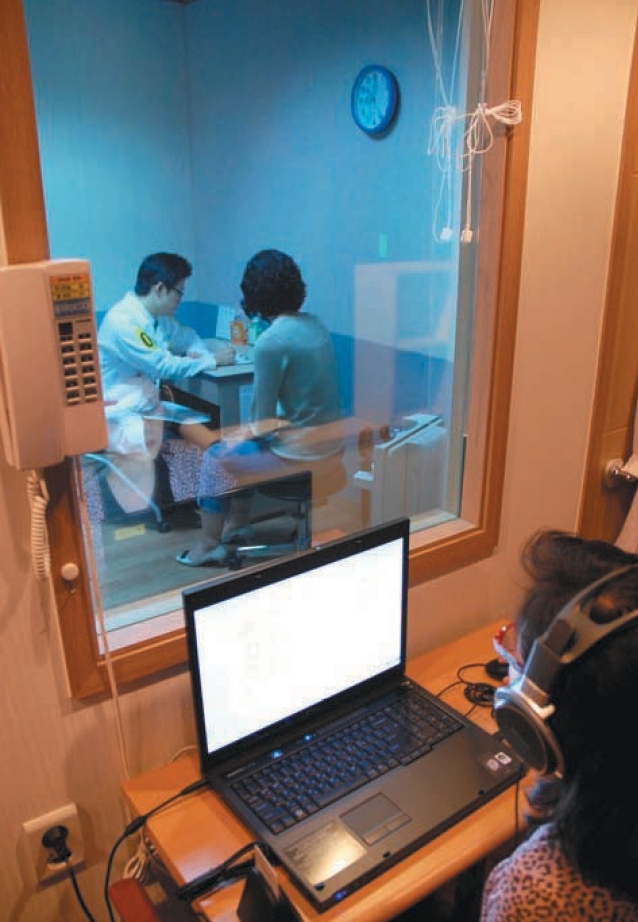
Scene of student's performance for the clinical performance examination with standardized patient viewed outside of oneway mirror room.

**Fig. 3:**
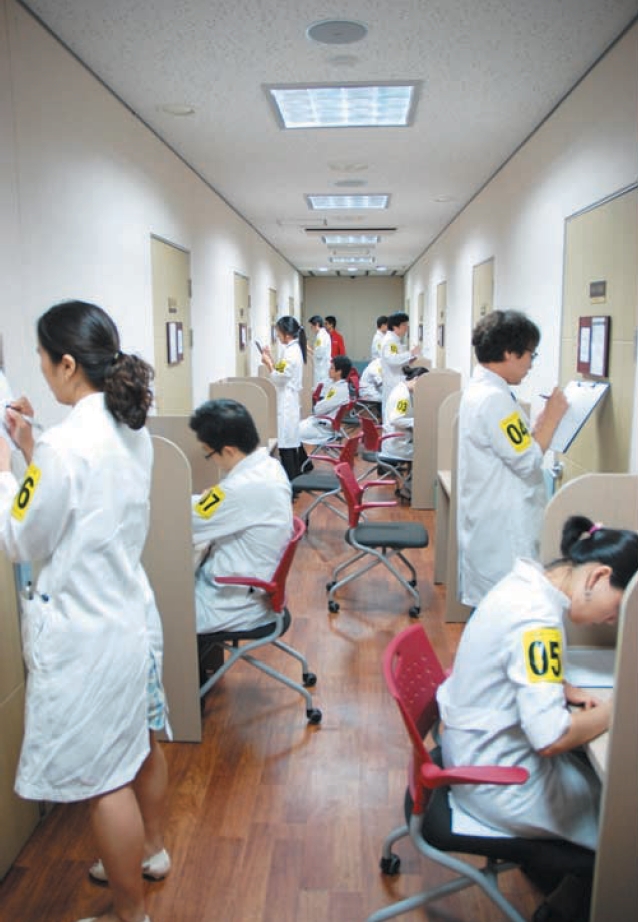
Examinees are answering the inter-station test during the inter-station interval.

**Fig. 4:**
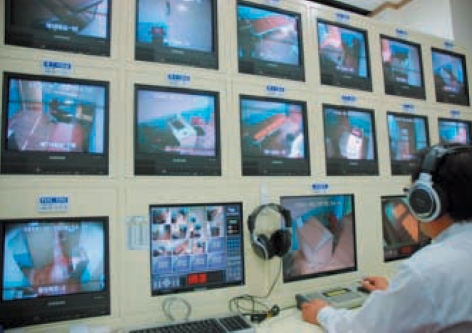
Monitoring room located in the clinical skill test center where every room is monitored for emergencies.

The dates available and the quota of applicants for each medical school were also announced. Applicants can choose the date of the test so that the right of choice by applicants was considered and any confusion arising by the crowding of applications at specific dates can be minimized. The test consisted of two types of evaluation, i.e., a clinical performance examination (CPX) with a standardized patient (SP) and objective structured clinical examination (OSCE). In the CPX, examinees examined the SP, after which their performance proficiency was rated by the SP. In the OSCE, a mannequin, model, or various equipment were used to rate the individual examinee's skill. The SP was a person trained to act as a real patient. The number of test items, score allotment, and test duration were summarized in Table 1. The same test was administered in three cycles a day: one cycle in the morning and two cycles in the afternoon, from 08:25 AM to 11:37 AM, from 12:00 MD to 15:12 PM, and 15:00 PM to 18:12 PM. Examinees moved to each of twelve sectors between the start bell and the ending bell and performed the tests provided (Figs. 1-4). The content of test items of the clinical skill test of the Korean Medical Licensing Examination in 2009 was listed in Table 2. Each single item of the 12 subjects determined was selected randomly from the item bank.

### Rating and setting of a passing score

The proctor (medical faculty member) and SP rate the examinees' proficiency for the OSCE and CPX respectively. The setting of a passing score was determined by two criteria. First, the applicant's total score should be equal to or greater than the sum of the passing scores of each of the 12 items that were set by the Committee for Reviewing of the Passing Score. Second, applicants' number of passing items should be equal to or greater than the number of passing items set by the Committee for Reviewing the Passing Score. A passing item meant that the applicant's score on an itemwas equal to or greater than the passing score set by the Committee for Reviewing the Passing Score. The passing score of each item and passing number of items were set by 12 medical faculty members of the Committee for Reviewing the Passing Score according to measurement theory from December 10 to 16, 2009. To earn a medical license, applicants should pass both the clinical skill test and written test. If an applicant passes only one test, it shall be effective until the next year so that the test passed shall be exempted in the next year. The number of raters is listed in Table 3.

### Results of the test

Out of 3,456 applicants who performed the first clinical skill test (September-December 2009), 3,289 examinees (95.2%) passed the test. Meanwhile, the written test of the 74th Medical Licensing Examination was administered from January 7 to 8, 2010 in six cities (Seoul, Busan, Daegu, Gwangju, Daejon, Jeonju). The application fee for the written test was 227,000 Korean won (200 US dollars). The three categories of subjects of the written test consisted of 115 items for general medicine, 365 items for specific areas, and 20 items for medical law. Out of 3,452 applicants, 3,349 examinees (97.0%) passed the test. Out of 3,469 applicants 3,439 applied to both the clinical skill test and written test, 17 examinees applied only to the clinical skill test and 13 applied only to the written test. Out of 167 examinees who failed the clinical skill test, 142 passed the written test. This means that the clinical skill test showed characteristics independent from the written test. The final passing rate for the 74th Korean Medical Licensing Examination was 92.9% (3,224/3,469), which was not significantly different from the result of the 73rd examination (93.6%).

### Special consideration for prevention of H1N1 influenza

To protect the applicants from H1N1 influenza epidemic infectious disease spreading in 2009, a variety of management plans were carried out [4]. The NHPLEB developed a close relationship with the nearby Regional Health Center. A fever detection camera was located in the front lobby of the NHPLEB and every person was screened for a fever. When the applicants' identification was verified, they were asked about any suspected symptoms of H1N1 influenza. If applicants were diagnosed or suspected to have H1N1 influenza, their test dates were allocated after their recovery or final diagnosis. During the whole period of the clinical skill test in 2009, two applicants were diagnosed with or suspected to have H1N1 influenza. They were allowed to apply for another test date.

## CONCLUSION

Since the 2009 clinical skill test was the first trial for the Medical Licensing Examination in Korea, there was a deal of great tension among and heavy workload for the staff of NHPLB and the participating faculty members and standardized patients. Nevertheless, all processes were satisfactory for continuing the trial. The opinions of examinees and raters will be analyzed and also published soon. Introducing the clinical skill test motivates every medical school in Korea to provide a clinical skill test lab and programs for OSCE and CPX to their students. Also, most students will do their best to perform clinical skills precisely. Korea is the first country to introduce the clinical skill test in Asia. I believe that this is the opening of a new era and environment that emphasizes performance and attitude in addition to knowledge in medical education in Korea. The NHPLB will do its best to improve performance test and to extend it to other health personnel licensing examinations.

## Figures and Tables

**Table 1:**
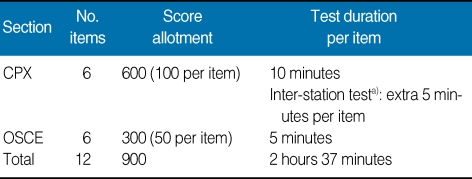
Number of test items, score allotment, and test duration

CPX, clinical performance examination; OSCE, objective structured clinical examination. ^a)^Test to record the diagnostic and therapeutic plan after examining a standardized patient during the inter-station interval.

**Table 2:**
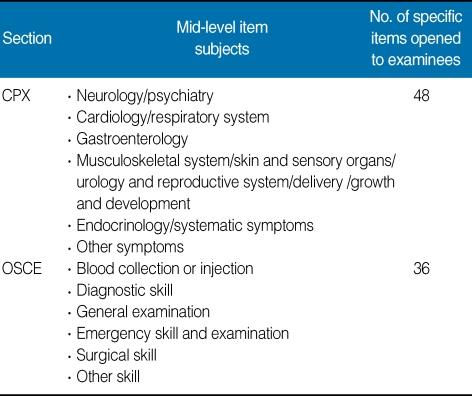
Subjects of test items of the clinical skill test of the Korean Medical Licensing Examination in 2009

CPX, cinical performance examination; OSCE, objective structured clinical examination.

**Table 3:**
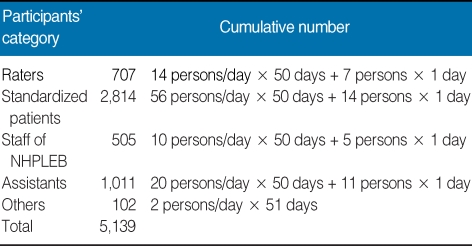
Cumulative number of raters and staff members who worked for the clinical skill test of the Korean Medical Licensing Examination (2009)

NHPLEB, National Health Personnel Licensing Examination Board.
